# Early Results of an Innovative Scalable Digital Treatment for Diabetes Distress in Families of School-Age Children with Type 1 Diabetes

**DOI:** 10.3390/children11101169

**Published:** 2024-09-26

**Authors:** Susana R. Patton, Jessica S. Pierce, Nicole Kahhan, Matthew Benson, Mark A. Clements, Larry A. Fox

**Affiliations:** 1 Center for Healthcare Delivery Science, Nemours Children’s Health, Jacksonville, FL 32207, USA; 2 Center for Healthcare Delivery Science, Nemours Children’s Health, Orlando, FL 32827, USA; jessica.pierce@nemours.org; 3 Division of Psychology, Nemours Children’s Health, Jacksonville, FL 32207, USA; nicole.kahhan@nemours.org; 4 Division of Endocrinology, Nemours Children’s Health, Jacksonville, FL 32207, USA; matthew.benson@nemours.org (M.B.);; 5 Division of Endocrinology, Children’s Mercy Hospital and Clinics, Kansas City, MO 64108, USA; maclements@cmh.edu

**Keywords:** diabetes, psychosocial, distress, treatment, parent, children, mHealth

## Abstract

Objective: This paper reports on the initial outcomes of a new mHealth intervention to reduce diabetes distress (DD) in families of school-age children living with type 1 diabetes (T1D) entitled, ‘Remedy to Diabetes Distress’ (R2D2). Methods: We randomized 34 families (mean child age = 10 ± 1.4 years; 53% male, 85% White, mean HbA1c = 7.24 ± 0.71%) to one of three delivery arms differing only by number of telehealth visits over a 10-week period: zero visits = self-guided (SG), three visits = enhanced self-guided (ESG), or eight visits = video visits (VV). All families had 24 × 7 access to digital treatment materials for 10 weeks. We examined the feasibility and acceptability of R2D2. We used the Problem Areas in Diabetes-Child (PPAIDC and PAIDC, parent and child, respectively) to examine treatment effects by time and delivery arm. We performed sensitivity analyses to characterize families who responded to R2D2. Results: It was feasible for families to access R2D2 mHealth content independently, though attendance at telehealth visits was variable. Parents and children reported high satisfaction scores. There were significant pre-post reductions in PPAIDC (*p* = 0.026) and PAIDC (*p* = 0.026) scores but no differences by delivery arm. There were no differences in child age, sex, race, or pre-treatment HbA1c for responders versus non-responders, though families who responded reported higher PPAID-C scores pre-treatment (*p* = 0.01) and tended to report shorter diabetes duration (*p* = 0.08). Conclusions: Initial results support the acceptability and treatment effects of R2D2 regardless of the frequency of adjunctive virtual visits. Characterizing responders may help to identify families who could benefit from R2D2 in the future.

## 1. Introduction

Daily management of type 1 diabetes consists of numerous complex, time-consuming, and relentless tasks for children living with this medical condition and their families [1]. The regimen involves a combination of accurate carbohydrate measurement, healthy eating, glucose monitoring, insulin administration, and physical activity [1]. Because of the specific knowledge, skills, and resources required to optimally manage type 1 diabetes, school-age children need help from their parents to complete daily type 1 diabetes care, and the burden of these ongoing demands can leave both parents and children vulnerable to feelings of diabetes distress (DD) [2,3].

Diabetes distress (DD) is an emotional and behavioral condition characterized by symptoms of fear, sadness, grief, anger, and in severe instances, diabetes burnout [4,5]. Additionally, persons experiencing DD may report challenges keeping up with type 1 diabetes self-care responsibilities and the use of less adaptive coping strategies (e.g., guessing an insulin dose to avoid checking glucose levels or skipping an insulin bolus altogether) [4,5]. DD is not the same as depression because it is not a mood disorder and because living with or caring for someone living with type 1 diabetes is the primary source or cause of DD [6].

DD is known to be common in school-age children with type 1 diabetes and their parents. In one published study examining DD in 8–12-year-olds, about 40% of children reported feelings of DD [4], and up to 33% of parents reported DD [4,7]. Cross-sectional studies suggest an association between higher child and parent DD and higher glycated hemoglobin (HbA1c) levels in school-age children living with type 1 diabetes [4,8,9,10,11]. There are also data indicating parent DD may be a stronger predictor of child HbA1c than the duration of type 1 diabetes or using an insulin pump [8], which may suggest reducing parent and child DD could be another pathway to helping families achieve their child’s HbA1c target.

Our group has been studying DD in families of school-age children living with type 1 diabetes with a long-term goal to design, test, and disseminate an intervention to help families reduce their feelings of DD. In a previous pilot, we tested an eight-session video-based telehealth-delivered intervention in 16 parents of school-age children with type 1 diabetes [12]. Parents reported a significant reduction in DD pre- to post-treatment (*p* = 0.01, d = 0.71) and there was a trend for a 0.78% reduction in child HbA1c (*p* = 0.06, d = 1.06) [12]. However, there remained a gap in available psychosocial interventions targeting DD in both parents and school-age children living with type 1 diabetes. Therefore, building on our existing parent-focused intervention, we used crowdsourcing methodology to enlist the help of parents of school-age children with type 1 diabetes in designing a scalable intervention to target DD in children and parents [13]. Through a series of open-ended questions posed to families over 20 weeks, parents characterized the challenges families face when managing type 1 diabetes, provided feedback on treatment strategies and tools to incorporate into our new treatment program, informed specific treatment content by sharing type 1 diabetes ‘life hacks’, personal experiences, and helpful coping strategies, and shaped our design of the new treatment’s format and appearance. Our crowdsource participants favored an mHealth treatment approach to promote family access and limit the impact of traditional barriers to in-person treatment, including travel, childcare, time away from work/school, and fear of public stigma [14,15]. However, because a fully self-guided mHealth delivery approach would be a complete departure from our previous, successful video-based telehealth intervention, we planned a small pilot to examine and compare the feasibility, acceptability, and treatment effects of a fully self-guided mHealth approach and two delivery approaches that combined mHealth with either three or eight video-based telehealth sessions. 

Here, we report the outcomes of our pilot examining the three treatment approaches for our novel intervention targeting DD in parents and school-age children living with type 1 diabetes. We also describe the early results of sensitivity analyses conducted to identify the characteristics of families who may respond to our new intervention versus families who may not respond. 

## 2. Materials and Methods

### 2.1. Participants and Procedures

Eligible families were English-speaking and had a child between 8 and 12 years old who was diagnosed with type 1 diabetes. We recruited families from two pediatric diabetes clinics in the southeast and midwestern regions of the United States. We obtained approval for our pilot from the Institutional Review Board at Children’s Mercy Hospital and Clinics (IRB#STUDY00002337) before enrolling families. We conducted the pilot trial under a SMART IRB agreement with Nemours Children’s Health in a reliance role. 

From parents, we obtained informed consent and permission for their child to participate using IRB-approved verbal consent procedures. We also obtained assent from children using approved verbal assent procedures. Following informed consent and assent, parents and children completed online surveys, provided at least two weeks of the child’s personal continuous glucose monitoring (CGM) and insulin pump data, and children completed a validated at-home HbA1c kit [16]. We compensated parents and children $40 for completing the pre-treatment study visit. Then, we randomized families to one of three different delivery arms of our novel mHealth intervention, called Remedy to Diabetes Distress (R2D2). We used variable block randomization to balance the number of families per treatment delivery arm [17]. Research coordinators obtained each family’s randomization assignment from a blinded master list. Once assigned to their treatment arm, all families received an email containing directions for how to access the mHealth treatment content. Families assigned to one of the treatment delivery arms that included video-based telehealth sessions also received information on how to schedule these visits. Families participated in the treatment phase of the pilot for 10 weeks. Once they completed the treatment phase, families completed the post-treatment study visit which included online surveys, gathering CGM and insulin pump data, and another at-home HbA1c kit. We compensated parents and children $40 for completing the post-treatment study visit and families ended participation in the pilot. 

### 2.2. Intervention 

R2D2 uses components of cognitive behavior therapy, mindfulness, and behavioral activation to help children and parents learn new strategies to manage feelings of DD [12,18,19,20]. Specifically, through eight mHealth modules, parents and children learn how to identify and reframe negative thoughts related to type 1 diabetes; use mindfulness and acceptance to manage feelings of anger, sadness, fear, or grief related to type 1 diabetes; and, add new activities to their daily lives to improve their mood, promote a sense of meaning, and foster a greater perception of mastery. R2D2 delivers its treatment content through a series of short (~90 s) videos and uses printable handouts and free, online resources to reinforce its main components. In addition, R2D2 includes a daily feelings tracker to help children and parents monitor their mood as well as a ‘quick tips’ feature that provides parents with a new strategy, helpful talkback, or ‘life hack’ related to living with type 1 diabetes each time they access the intervention. We designed R2D2 to be accessible to parents via our hospitals’ online patient portal applications (apps). 

### 2.3. Treatment Delivery Approaches

Families participated in R2D2 through one of three delivery approaches. In the fully self-guided (SG) arm, families had 24 × 7 access to the R2D2 mHealth treatment for 10 weeks. Parents and children had maximum flexibility in when, how often, and at what pace they wished to view the R2D2 treatment materials. Families in SG did not participate in video-based telehealth sessions, but parents did receive periodic text messages from the study team to let them know how much time they had left to access the R2D2 treatment materials to encourage engagement. In the enhanced self-guided (ESG) arm, families had 24 × 7 access to the R2D2 mHealth treatment for 10 weeks. In addition, parents and children met with an R2D2 counselor for a 30 min video-based telehealth session during weeks 2, 4, and 6 of the treatment period. Parents and children could use these video-based telehealth sessions to ask questions about the R2D2 treatment materials and/or to practice new skills with coaching from the R2D2 counselor. In the video visit (VV) arm, families had 24 × 7 access to the R2D2 mHealth treatment for 10 weeks. In addition, parents and children met with an R2D2 counselor for 8 video-based telehealth sessions (30–45 min per session). Like the ESG, parents and children could use the video-based telehealth sessions to ask questions about the R2D2 treatment materials and/or to practice new skills with coaching from the R2D2 counselor. However, we also allowed for these telehealth visits to run longer in case families did not access R2D2 on their own and were waiting for the R2D2 counselor to present the treatment materials. 

### 2.4. Measures

Demographics: We collected all demographic information on families using an electronic survey. In addition, parents reported on whether their children used an insulin pump or CGM for their daily diabetes care. 

Parent Problem Areas in Diabetes-Child (PPAID-C): We used the PPAID-C to measure parent’s DD. The PPAID-C is a validated 16-item survey. Item responses follow a six-point Likert scale (1 = not a problem to 6 = big/serious problem), which can be summed to yield a total score (possible range: 16–96) with higher scores indicating greater perceived distress [4].

Problem Areas in Diabetes-Child (PAID-C): We used the PAID-C to measure children’s DD. The PAID-C is a validated 11-item survey. Item responses follow a six-point Likert scale (1 = not a problem to 6 = big/serious problem), which can be summed to yield a total score (possible range: 11–66) with higher scores indicating greater distress [4].

Treatment Satisfaction: We used study-specific surveys to rate parent and child treatment satisfaction. Parents completed a version that included 14 items, while children completed a version with 8 items. Parents and children each responded to items using a five-point Likert scale (0–4), with higher scores reflecting greater satisfaction. 

Hemoglobin A1c (HbA1c): We used a validated finger-stick home HbA1c kit at pre- and post-treatment to measure children’s average glycemic levels [16]. We used a central laboratory and automated high-performance liquid chromatography (reference range 4.0–6.0% [20–42 mmol/mol]; Tosoh Corporation, San Francisco, CA, USA) for these analyses [21].

### 2.5. Statistical Analysis

We conducted all study analyses on SPSS version 27 and applied an alpha of *p* < 0.05 to signify statistical significance. We calculated descriptive statistics for our demographic and outcome variables. We used repeated measures analysis of variance (ANOVA) with the treatment delivery arm (SG, ESG, VV) as between-subject variables, time as a within-subject variable, and parent and child PAID scores as the dependent variable. To examine the characteristics of families who responded versus those who did not respond to our R2D2 treatment, we used the SEM small effect formula:

[1*(SD*√(1 − α)],(1)

as an indicator of minimal clinically important differences (MCID) [22]. We calculated MCIDs separately for parents and children. We defined treatment responders as persons with a ≥1 MCID decrease in PAID scores pre- to post-treatment. Alternatively, we defined treatment non-responders as persons with a ≥1 MCID increase or no change in PAID scores pre- to post-treatment. We then categorized parent–child dyads based on whether both the parent and child responded (double responders), neither the parent nor child responded (non-responders), or one person in the parent–child dyad responded (mixed responders). We used separate ANOVA or chi-square to examine the characteristics of our double responders, non-responders, and mixed responders. 

## 3. Results

Table 1 describes pre-treatment demographics and child type 1 diabetes data for the full sample and separately for families randomized to each treatment delivery arm. Of the 42 families enrolled, 34 completed the pre-treatment visit, 34 were randomized, and 33 completed the post-treatment visit (19% attrition prior to treatment; 3% attrition after treatment). Most participating parents were mothers. The most frequent reason parents gave for withdrawing from the trial was lack of time (n = 5). It was also common for parents to passively withdraw by not responding to study staff messages (n = 4).

### 3.1. Feasibility

We randomized 13 families to the SG, 11 to the ESG, and 10 to the VV treatment arm. Eighty-one percent of families randomized to ESG completed all three video-based telehealth visits while only 20% of families randomized to VV completed all eight video-based telehealth visits. The mean number of video-based telehealth visits completed for ESG families was 2.7 ± 0.6 and 4.6 ± 2.2 for VV families. We also captured the number of minutes families interacted with R2D2 mHealth treatment through their hospital app. On average, families interacted with the R2D2 mHealth content 38.51 ± 40.52 min (range: 1.5 to 188.2 min) during the 10-week trial and there was no difference between groups (SG, ESG, or VV) for average minutes (see Table 1). 

### 3.2. Acceptability

Overall, parents reported an item mean score of 3.75 ± 0.36 and children reported an item mean score of 3.23 ± 0.83 on their respective post-treatment satisfaction surveys. Parents’ scores corresponded to a 94% satisfaction score, while children’s scores corresponded to an 81% satisfaction score. There were no group differences in parent or child satisfaction scores (Table 2). One item per survey asked parents (item 14) or children (item 8) if they would recommend R2D2 to other families of children living with type 1 diabetes. A total of 94% of parents responded with ‘true’ or ‘very true’ to this item, while 85% of children responded with ‘true’ or ‘very true’ to this item. Again, we observed no group differences in how parents or children responded to this item (see Table 1). 

### 3.3. Pre-Post Treatment Outcomes

Repeated-measures ANOVA results indicated the main effects of time for parent and child distress scores. Specifically, both parents (η_p_^2^ = 0.22, *p* = 0.008) and children (η_p_^2^ = 0.16, *p* = 0.01) reported significant reductions in DD pre- to post-treatment. We observed no main effects for group (SG, ESG, or VV) for parent and child distress scores. We also did not find a significant main effect for time or group for child HbA1c. 

### 3.4. Sensitivity Analyses

In sensitivity analyses, 14 parent–child dyads qualified as double responders (42%), 12 dyads qualified as mixed responders (36%), and 8 dyads qualified as non-responders (24%). Double responders reported significantly higher pre-treatment parent distress scores than mixed responders (*p* = 0.02) and non-responders (*p* = 0.005). There was a trend suggesting double responders reported a shorter duration of type 1 diabetes than mixed responders (*p* = 0.08). We found no significant differences between responders and non-responders with respect to child age, child pre-treatment distress scores, and child HbA1c. We found no significant differences in the number of responders versus non-responders by treatment delivery arm (see Table 2). Finally, with respect to other treatment factors, we found no associations between minutes of interacting with the R2D2 mHealth content and parent and child post-treatment distress scores. We found no associations between parents’ pre- or post-treatment distress scores and their treatment satisfaction scores. In contrast, children’s pre- and post-treatment distress scores were both negatively associated with their treatment satisfaction score, suggesting children with higher pre- and post-treatment distress tended to report less treatment satisfaction (See Table 3).

## 4. Discussion

DD is common in school-age children with type 1 diabetes and their parents and is related to higher glycemic levels and lower treatment engagement [4,8,9,10,11]. Therefore, there is a strong rationale for developing scalable treatment programs to help families of school-age children with type 1 diabetes reduce their feelings of DD. Building off our existing video-based telehealth intervention that showed initial efficacy in treating DD in parents of school-age children [12], we collaborated with parent stakeholders to develop R2D2 as an mHealth intervention to target DD in both school-age children with type 1 diabetes and their parents. Here, we report the results of a pilot trial examining the feasibility, acceptability, and treatment effects of a fully self-guided mHealth approach of R2D2 (SG) compared to two delivery approaches that combined our R2D2 mHealth content with either three (ESG) or eight video-based telehealth sessions (VV).

Our results indicate that R2D2 shows a treatment signal for reducing DD for both parents and children, as both experienced reductions in DD from pre- to post-treatment across the three treatment arms. However, our results also show no difference in treatment signal based on the treatment arm, suggesting the self-guided R2D2 approach may be similarly efficacious in reducing DD in parents and children as an approach that combines the mHealth treatment content with three or eight video-based telehealth sessions. There is evidence from the general literature supporting the acceptability and efficacy of using a self-guided mHealth approach to treat depression and anxiety [23,24,25]. Additionally, one study has reported initial success in reducing DD among adults living with either type 1 diabetes or type 2 diabetes using an mHealth approach [26], and there are two trials underway testing web-based [27] and text-message-based [28] interventions to reduce DD in adolescents living with type 1 diabetes. Nonetheless, we believe we are the first to report on early treatment effects of a self-guided mHealth approach to treat DD in families of school-aged children living with type 1 diabetes. Moreover, we assert that an mHealth approach for teaching parents and children evidence-based strategies to reduce DD has several advantages including the potential to reduce barriers to traditional, in-person treatment (e.g., travel, transportation, time away from everyday responsibilities, fear of public stigma) [14,15] and the opportunity for greater ecological validity because parents and children can learn and practice new strategies to reduce DD in the context of their everyday lives where they are likely to experience DD [29]. 

Like our primary analyses examining the treatment signal of R2D2, we found R2D2 acceptability was generally high with no differences in acceptability among the three treatment arms, indicating that families found the self-guided intervention acceptable regardless of whether they also received additional video-based telehealth sessions. Parents favored the intervention slightly more than their children, which may, in part, be the result of our design methods. We only involved parents in the intervention development phase of R2D2. Future studies should consider obtaining feedback on intervention content and format directly from school-age children as that may be one method to increase their perceptions of acceptability. 

With respect to the feasibility of R2D2, a higher percentage of families who were randomized to ESG completed all three video-based telehealth visits (81%) compared to the percentage of families randomized to VV who completed all eight video-based telehealth visits (20%). This suggests completion of, and engagement with, the three-session ESG treatment approach may be more feasible than the eight-session VV approach. It is notable, however, that with a 10-week treatment period, families assigned to the VV treatment approach also had fewer spare weeks to make up missed sessions than families in the ESG approach. Therefore, it is possible with a longer treatment period, the VV approach could appear more feasible, as families would have more weeks to make up missed sessions. When examining the number of minutes that families interacted with R2D2 treatment content through their hospital app, it is notable that there were no group differences. This suggests the addition of video-based telehealth visits did not affect how much families opted to interact with the R2D2 mHealth content. We also think it is notable that R2D2 appeared helpful in reducing DD in families with a relatively low treatment dose (overall mean = 38.51 ± 40.52 min). This average time could equate to families watching all the R2D2 videos (20.6 min of content) at least one time as well as accessing other online materials to reinforce the treatment content. In a future larger trial, we will continue to explore families’ experiences while interacting with the R2D2 mHealth content. However, these initial data suggest the potential feasibility and scalability of our R2D2 intervention. 

Finally, we conducted sensitivity analyses to characterize the families who appeared to be responding to our R2D2 treatment [30]. We did not find any differences in child age, sex, race, or pre-treatment HbA1c for responders versus non-responders, though parents who responded had higher DD scores pre-treatment and children tended to have shorter diabetes duration. These findings highlight the importance of routine screening for DD and timely treatment referral for all families living with type 1 diabetes, as those who are already experiencing distress and those who are newly adjusting to life with type 1 diabetes both appear likely to benefit from treatment. Though we found non-significant associations between minutes interacting with the R2D2 mHealth treatment and post-treatment DD in parents and children, there was wide variability among families in minutes interacting with the mHealth content, which likely reduced the power. In a larger trial, it might be important to re-examine these associations because even though it was not significant, the association for children was moderate in strength and suggested more time interacting with the R2D2 mHealth content may relate to less post-treatment DD. In correlations relating parent and child DD scores to their level of treatment satisfaction, there were no associations for parents but there were significant negative associations for children, which may further underscore the value of seeking child feedback to enhance our R2D2 treatment content and delivery approach.

Limitations of our small pilot trial include its sample size, its disproportionate representation of White and Non-Hispanic families, its inclusion of children with well-controlled HbA1c levels, its limited father or other caregiver participation, the possibility that we were underpowered in our analyses, and the absence of a standard care group. Strengths include its randomized design, its innovation in testing an mHealth treatment targeting DD in families of school-age children, its comparison of treatment effects for families who completed R2D2 in a self-guided format versus a format that included varying numbers of video-telehealth visits to reinforce the treatment content, and its use of validated outcome measures. 

## 5. Conclusions

In this R2D2 pilot, we observed a reduction in DD in school-age families after 10 weeks of exposure to an innovative mHealth treatment program. We found no differences in treatment effects for families who completed our R2D2 mHealth treatment in a fully self-guided approach versus an approach that included three or eight video-based telehealth sessions. Also, we found no differences in parent and child acceptability ratings for our three R2D2 delivery approaches, though early results suggest it may be less feasible to use the delivery approach that included eight video-based telehealth sessions. In sensitivity analyses, results suggest R2D2 could particularly benefit families experiencing higher levels of distress and/or families of children with recent-onset type 1 diabetes. Our next step in this line of research is to conduct a larger, randomized trial comparing our R2D2 mHealth treatment program to usual care to further test its efficacy in reducing DD in families of school-age children and to examine its potential to help children achieve and maintain their target glycemic levels [31].

## Figures and Tables

**Table 1:** Sample characteristics and outcomes for the overall sample and by delivery arm.

	Overall n = 34	VV n = 10	ESG n = 11	SG n = 13	*p* ^†^
Child age, yr (M ± SD)	10.1 ± 1.4	10.2 ± 1.3	10.3 ± 1.7	9.8 ± 1.2	0.73
Type 1 diabetes duration, yr (M ± SD)	6.7 ± 2.8	6.7 ± 2.9	7.6 ± 2.2	5.9 ± 3.1	0.38
Pre-treatment HbA1c, % (M ± SD)	7.24 ± 0.71	7.38 ± 0.80	7.22 ± 0.47	7.16 ± 0.84	0.77
Child identified male, n (%)	16 (47)	5 (50)	3 (27)	8 (62)	0.24
Family identified:					0.53
White n (%)	31 (91)	10 (100)	9 (82)	12 (92)	
Black or African American n (%)	1 (3)	0	1 (9)	0	
More than one race n (%)	2 (6)	0	1 (9)	1 (8)	
Hispanic n (%)	2 (6)	1 (10)	1 (9)	0	
Insulin pump, n (%)	27 (79)	8 (80)	8 (73)	11 (85)	0.77
Open pump	9 (33)	3 (30)	6 (75)	0	0.004
Automated insulin delivery	18 (66)	5 (50)	2 (25)	11 (100)	
Continuous glucose monitor, n (%)	34 (100)	10 (100)	11 (100)	13 (100)	--
Parent age, yr (M ± SD)	42.2 ± 6.1	44.9 ± 7.0	40.6 ± 5.0	41.5 ± 6.0	0.25
Mother, n (%)	33 (97)	10 (100)	11 (100)	12 (92)	0.43
Pre-treatment PPAID-C (M ± SD)	46.4 ± 15.6	44.2 ± 16.5	45.2 ± 17.1	49.2 ± 14.3	0.72
Pre-treatment PAID-C (M ± SD)	32.3 ± 13.3	33.9 ± 14.4	30.3 ± 12.5	32.7 ± 13.9	0.82
Post-treatment PPAID-C (M ± SD)	41.3 ± 12.7	41.1 ± 14.0	41.9 ± 12.2	40.9 ± 13.0	0.98
Post-treatment PAID-C (M ± SD)	28.6 ± 11.2	29.7 ± 11.6	28.6 ± 12.0	27.8 ± 11.2	0.93
Parent satisfaction (item M ± SD)	3.75 ± 0.37	3.75 ± 0.27	3.91 ± 0.14	3.61 ± 0.52	0.19
Child satisfaction (item M ± SD)	3.23 ± 0.83	3.57 ± 0.81	3.41 ± 0.60	2.84 ± 0.89	0.08
Minutes interacting with R2D2 mHealth treatment content (M ± SD)	38.5 ± 40.5	42.3 ± 45.6	42.2 ± 53.7	32.5 ± 21.7	0.80

Note. M, mean; SD, standard deviation; yr, years; PPAID-C, Parent Problem Areas in Diabetes-Child; PAID-C, Problem Areas in Diabetes-Child; VV, virtual visit delivery approach; ESG, enhanced self-guided delivery approach; SG, self-guided delivery approach; ^†^ group comparisons for the three treatment delivery approaches.

**Table 2:** Characteristics of R2D2 responders, mixed responders, non-responders.

	Double Responders n = 14	Mixed Responders n = 12	Non-Responders n = 8	*p*
Child age, yr (M ± SD)	10.4 ± 1.1	9.7 ± 1.5	10.2 ± 1.6	0.43
Type 1 diabetes duration, yr (M ± SD)	2.6 ± 2.1 ^a^	4.6 ± 3.1	3.0 ± 2.5	0.06
Pre-treatment HbA1c, % (M ± SD)	7.18 ± 0.70	7.36 ± 0.89	7.19 ± 0.45	0.79
Post-treatment HbA1c, % (M ± SD)	7.31 ± 0.79	7.43 ± 0.98	7.24 ± 0.46	0.88
Pre-treatment PPAID-C (M ± SD)	55.4 ± 15.5 ^b,c^	42.2 ± 12.7	37.0 ± 12.1	0.01
Pre-treatment PAID-C (M ± SD)	36.0 ± 12.6	32.2 ± 14.9	25.7 ± 10.5	0.22
Post-treatment PPAID-C (M ± SD)	42.1 ± 11.6	39.2 ± 14.9	43.0 ± 12.2	0.79
Post-treatment PAID-C (M ± SD)	26.9 ± 11.0	28.0 ± 11.9	33.1 ± 10.8	0.49
Parent satisfaction (item M ± SD)	3.86 ± 0.26	3.69 ± 0.51	3.62 ± 0.31	0.35
Child satisfaction (item M ± SD)	3.18 ± 1.01	3.13 ± 0.67	3.51 ± 0.73	0.62
Minutes interacting with R2D2 mHealth treatment content (M ± SD)	34.2 ± 26.1	30.0 ± 40.6	58.8 ± 57.0	0.27
Insulin pump, n (%)	12 (86)	10 (83)	5 (62.5)	
Open pump, n (%)	5 (42)	1 (10)	3 (60)	0.23
Automated insulin delivery, n (%)	7 (58)	9 (90)	2 (40)	
*R2D2 delivery arm*:				0.43
Virtual Visit n (%)	4 (28)	2 (16.5)	4 (50)	
Enhanced self-guided n (%)	4 (28)	4 (33.5)	3 (37.5)	
Self-guided n (%)	6 (44)	6 (50)	1 (12.5)	

Note, ^a^ *p* = 0.06 for double responders vs. mixed responders; ^b^ *p* = 0.02 for double responders vs. mixed responders; ^c^ *p* = 0.005 for double responders vs. non-responders.

**Table 3:** Correlations between sample characteristics, treatment factors, and distress scores.

	Pre-Treatment PPAID-C	Pre-Treatment PAID-C	Post-Treatment PPAID-C	Post-Treatment PAID-C
Child age	0.32 ^†^	0.23	0.25	0.22
Parent age	−0.13	−0.02	0.13	0.05
Type 1 diabetes duration	0.11	−0.24	0.02	−0.37 *
Pre-treatment HbA1c	0.03	0.29	0.022	0.13
Post-treatment HbA1c	0.10	0.36 *	0.30	0.22
Minutes interacting with R2D2 mHealth content	−0.04	−0.18	0.19	−0.23
Parent satisfaction	0.07	0.24	−0.29	−0.02
Child satisfaction	−0.22	−0.50 **	−0.27	−0.42 *

Note, ^†^ *p* < 0.07, * *p* < 0.05, ** *p* < 0.001.

## Data Availability

Upon request, the authors will make raw data supporting the conclusions of this article available. The authors will consider requests to access the datasets on a case-by-case basis and subject to review and approval by the appropriate ethics board.
